# Parental Attitudes Towards Vaccination in Children with Inflammatory Bowel Disease: A Comparative Study

**DOI:** 10.3390/children13020238

**Published:** 2026-02-07

**Authors:** Svetlana I. Erdes, Ivan S. Samolygo, Mikhail P. Kostinov, Olga L. Lomakina, Ekaterina A. Yablokova, Anton S. Antishin, Albina S. Pestova, Viktoria S. Krikun, Yulia A. Drozdova, Elena V. Borisova, Marina A. Manina

**Affiliations:** 1 Department of Propaedeutics of Children’s Diseases, N.F. Filatov Clinical Institute of Children’s Health, Sechenov First Moscow State Medical University (Sechenov University), 119991 Moscow, Russia; erdes_s_i@staff.sechenov.ru (S.I.E.); antishin_a_s@staff.sechenov.ru (A.S.A.); pestova_a_s@staff.sechenov.ru (A.S.P.); krikun_v_s@staff.sechenov.ru (V.S.K.); drozdova_yu_a@staff.sechenov.ru (Y.A.D.); manina_m_a@staff.sechenov.ru (M.A.M.); 2 Laboratory of Vaccine Prevention and Immunotherapy of Allergic Diseases, Federal State Budgetary Scientific Institution «I. Mechnikov Research Institute of Vaccines and Sera», 105064 Moscow, Russia; kostinov_m_p@staff.sechenov.ru; 3 Department of Epidemiology and Modern Vaccination Technologies, Federal State Autonomous Educational Institution of Higher Education, Sechenov First Moscow State Medical University (Sechenov University), 119991 Moscow, Russia; 4 Center for the Treatment of Rheumatic Diseases, Children Morozov Children’s City Clinical Hospital, 119049 Moscow, Russia; lomakolga@mail.ru; 5 Department of Childhood Diseases, N.F. Filatov Clinical Institute of Children’s Health, Sechenov First Moscow State Medical University (Sechenov University), 119991 Moscow, Russia; yablokova_e_a@staff.sechenov.ru; 6 Department of Gastroenterology, Sechenov Center for Motherhood and Childhood, Sechenov First Moscow State Medical University (Sechenov University), 119991 Moscow, Russia; borisova_e_v@staff.sechenov.ru

**Keywords:** inflammatory bowel disease, children, vaccination, vaccine adherence, vaccination barriers, vaccine hesitancy

## Abstract

**Highlights:**

**What are the main findings?**

- Despite high pre-diagnosis vaccination rates (>93%), 78% of parents refuse further immunization after their child is diagnosed with IBD.
- Parents frequently misinterpret normal post-vaccination reactions as serious complications and rely on unverified internet sources due to a lack of proactive physician guidance.

**What are the implications of the main findings?**

- Vaccine hesitancy in this population is “acquired” rather than inherent, necessitating early educational intervention by gastroenterologists at the time of diagnosis.
- Proactive counseling and targeted educational programs are essential to correct safety misconceptions regarding immunosuppression and restore vaccination adherence.

**Abstract:**

**Objective:** To evaluate parental attitudes towards vaccination in children with inflammatory bowel disease (IBD), assess the level of adherence to immunization schedules, and identify key barriers hindering vaccination. **Materials and Methods:** A comparative survey was conducted involving 215 respondents, divided into an IBD group (109 parents of children with IBD) and a control group (106 parents of healthy children). The majority of respondents were mothers (96%) with higher education (81% and 79%, respectively) residing in a major metropolitan area. We assessed demographic data, vaccination history of both children and parents, sources of medical information, and reasons for vaccine refusal. **Results:** Routine vaccination coverage in children under 6 years of age was high and comparable in both groups (>93%). The majority of parents in the IBD group (*n* = 68; 62%) expressed a positive attitude towards vaccination. However, following the onset of IBD, only 24 (22%) continued vaccination, while 85 (78%) reported a categorical refusal to continue immunization. It was found that parents tend to misinterpret normal post-vaccination reactions as vaccine complications. A significant factor contributing to refusal is the lack of information from attending physicians and reliance on the Internet as a primary information source. Additionally, low rates of adult revaccination were noted, with over 30% of parents in both groups not being vaccinated in adulthood. **Conclusions:** The low vaccination rate in children with IBD after disease onset is driven not by initial anti-vaccination sentiment, but by acquired fears and a lack of professional communication from primary care providers and specialists. Improving immunization coverage requires the active implementation of educational programs for parents regarding vaccine safety during immunosuppressive therapy, as well as the development of specific guidelines for attending physicians.

## 1. Introduction

Inflammatory bowel disease (IBD) comprises a group of chronic, immune-mediated gastrointestinal disorders with systemic manifestations [1,2]. The management of IBD relies heavily on long-term immunosuppressive therapy, which inevitably compromises the host’s defense mechanisms against infectious diseases [3,4]. Several studies have demonstrated that patients with IBD are at an increased risk of developing infectious diseases [5]. Timely preventive vaccination is a safe and effective strategy to mitigate the burden of infections in patients with IBD [5]. Given the rising incidence of pediatric IBD and the increasing use of biological therapies, enhancing knowledge regarding vaccination in this population is crucial for primary care physicians, pediatric gastroenterologists, and infectious disease specialists [6]. However, persistent fears and myths surrounding vaccination in IBD patients—among both parents and healthcare providers—contribute to suboptimal vaccination rates. For instance, a data analysis of 1722 IBD patients by García-Serrano et al. revealed that vaccination coverage remained below 65% for all vaccines studied. Furthermore, only 10.9% of patients with IBD continue vaccination after the onset of the disease [7]. The primary reasons for incomplete vaccination in children with IBD include concerns regarding the safety of vaccines in this patient population [8,9]. In the Russian Federation, childhood vaccination is carried out according to the National Immunization Schedule and is provided free of charge through public funding [10]. Studies have shown that parents of children with IBD are three times more likely to express concern about vaccine safety compared to parents of healthy controls [11]. Another significant factor contributing to incomplete vaccination is the lack of awareness among primary care physicians regarding specialized vaccination algorithms and the feasibility of continuing immunization after IBD onset [12]. Surveys of physicians and parents indicate that poor continuity of care and insufficient collaboration between different levels of the healthcare system result in low awareness and missed vaccination opportunities [6,13]. While educational campaigns for physicians have been shown to increase vaccination rates, they often fail to achieve optimal coverage levels [14,15]. Notably, the fear of disease exacerbation (flare-ups) remains one of the principal barriers to vaccination among parents of children with IBD [16].

To the best of our knowledge, this study represents the most extensive comparative analysis to date in the Russian Federation, directly contrasting the attitudes of parents of children with IBD against a control group of healthy children. Unlike previous studies, often limited by small sample sizes, the novelty of this research lies in its comprehensive approach: beyond assessing general attitudes, we evaluated the parents’ specific knowledge and their interpretation of adverse events following immunization. We demonstrate for the first time in this population that vaccine hesitancy is primarily “acquired” post-diagnosis rather than pre-existing, driven by a lack of safety information regarding immunosuppression. Furthermore, this study uniquely incorporates the vaccination status of the parents themselves, highlighting broader family patterns in immunization behavior.

The aim of this study was to assess vaccination adherence and attitudes among parents of children with IBD compared to a control group, as well as to identify the main barriers to immunization in order to develop strategies for improving vaccination coverage.

## 2. Materials and Methods

**Figure 1:**
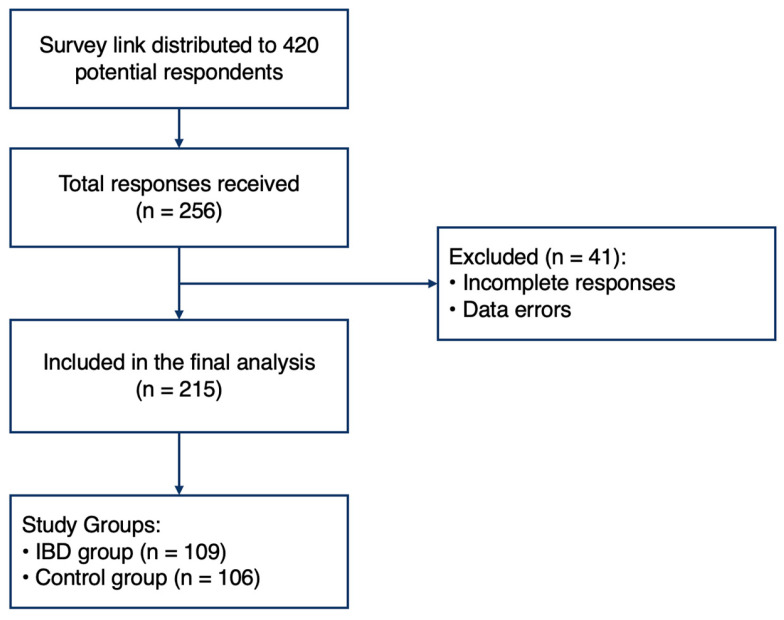
Flowchart of participant selection.

This cross-sectional, comparative study was based on an electronic survey of parents of children with a confirmed diagnosis of Inflammatory Bowel Disease (IBD) and parents of a control group. A total of 215 participants were included in the study: 109 parents of children with IBD and 106 parents of children in the control group. The process of participant recruitment and selection is illustrated in Figure 1.

The diagnosis of IBD was established in accordance with the ESPGHAN “Porto” criteria (2014) [2]. The survey of parents of children with IBD was conducted at I.M. Sechenov First Moscow State Medical University between September 2024 and November 2025. Data from parents whose children’s diagnosis was questionable or unconfirmed were excluded from the analysis. The control group consisted of parents of children belonging to health groups 1 and 2 (healthy children). The study was approved by the Local Ethics Committee of Sechenov First Moscow State Medical University (Sechenov University), Moscow, Russian Federation (Protocol No. 18-24, dated 18 July 2024). Respondents were invited to participate in the study via an electronic questionnaire. The survey was completely anonymous and did not collect any personal identifying information. The questionnaire was developed by the authors. The survey for parents of children with IBD consisted of three main sections (19 questions), while the survey for the control group also contained three sections but with fewer questions (15 questions). The sections covered the following topics:

- Section S1: General data. This section included questions regarding the child’s age, gender, and diagnosis (Ulcerative Colitis (UC) or Crohn’s Disease (CD)). It also assessed the parents’ (mother and father) education level and the child’s current primary activity (preschooler, school student, or university student).
- Section S2: Parental attitude toward vaccination. This section contained questions about the parents’ personal attitude toward vaccination, their history of vaccination in adulthood, the occurrence of any adverse events following immunization, and the specification of such events if present.
- Section S3: Child vaccination. This section included questions on early childhood vaccination history, the occurrence of adverse events following immunization (with specification of the events), and the vaccines associated with adverse reactions. Additionally, the IBD-specific questionnaire included items regarding the continuation of vaccination after the diagnosis (CD or UC) and the parents’ search for information on vaccination feasibility post-diagnosis. The full version of the questionnaire is available in the Supplementary Materials.

Statistical analysis was performed using Microsoft Excel 2024 and jamovi 2.7.5. The normality of distribution for quantitative variables was assessed using the Shapiro–Wilk test. Quantitative data with non-normal distribution are presented as medians with interquartile ranges (IQR). Qualitative data are presented as absolute values and percentages (%). Given the non-normal distribution of most variables, the Mann–Whitney U test was used to assess the statistical significance of differences between groups for quantitative data. For qualitative data, Pearson’s Chi-squared test (χ^2^) was applied, with the calculation of odds ratios (OR) and 95% confidence intervals (95% CI). In cases where expected frequencies were low, Fisher’s exact test was applied to ensure statistical accuracy. Binary logistic regression analysis was performed for selected categorical variables. A *p*-value of <0.05 was considered statistically significant.

## 3. Results

### 3.1. Socio-Demographic Characteristics of Parents of Children with IBD and Healthy Children

The baseline characteristics of the study participants are presented in Table 1. The vast majority of respondents (96%) were women—specifically, mothers of children with IBD and mothers of healthy children (*p* = 0.968). The median age of mothers in the IBD group was 35 years (IQR 27–50), whereas the median age of mothers in the control group was lower, at 30 years (IQR 25–45). All participants (mothers in both the IBD and control groups) were urban residents living in a large metropolis. Among the surveyed mothers, the proportion of those with completed higher education was predominant in both groups, accounting for 81% in the IBD group and 79.2% in the control group (Table 1). Meanwhile, the share of mothers without higher education (completed secondary specialized education or incomplete higher education) was 19.3% and 21% in the IBD and control groups, respectively. Among fathers, the proportion of individuals with completed higher education was lower compared to mothers in both groups, at 68% and 74.5%, respectively (Table 1). Consequently, there was a higher percentage of fathers without higher education—25.5% in the IBD group and 32.9% in the control group. The overwhelming majority of surveyed mothers were permanently employed at the time of inclusion in the study: 93% and 96% in the IBD and control groups, respectively. Among the surveyed parents of children with IBD, 11 (10%) individuals had also been diagnosed with IBD.

### 3.2. Demographic Characteristics of Children with IBD and Healthy Children

The age and gender distribution of children with IBD and healthy children was as follows: the IBD group included 59 girls and 50 boys, while the group of healthy children included 56 girls and 50 boys (*p* = 0.78). The median age of children with IBD was 13 years (IQR 10–16), whereas in the control group, it was 12 years (IQR 9–15). The median age at IBD onset was 11 years (IQR 8–15). Data regarding the children’s primary activity at the time of study inclusion are presented in Table 1. As shown in Table 1, the majority of children in the IBD group were full-time school students (84; 77%) at the time of the survey. A similar proportion (81; 76%) of participants in the control group were also full-time school students. Among all school-aged participants, home-based education was reported exclusively for children in the IBD group (5; 4.6%). Conversely, a higher number of participants in the control group attended preschool institutions (10; 9.4%) compared to children in the IBD group (2; 1.8%).

### 3.3. Respondents’ Attitude Toward Vaccination

During the survey, respondents were asked to indicate their attitude toward vaccination by selecting one of three provided options: “Positive; vaccination is necessary to build lasting immunity against infections,” “Skeptical/With distrust; I think vaccination is needed, but I worry about the consequences,” or “Negative; I believe vaccination can be potentially dangerous to health.” The responses did not differ significantly between the groups (*p* = 0.27). The majority of study participants expressed a positive perception of vaccination: 62% (68 individuals) of parents of children with IBD and 73% (77 individuals) of parents of healthy children (Table 1). Parents were two to three times less likely to express skepticism about vaccination than those who expressed a positive attitude: 24 (23%) parents in the control group and 33 (30%) in the IBD group. The smallest number of respondents in both groups reported a negative attitude toward vaccination: 5% (5 individuals) in the control group and 7% (8 individuals) in the IBD group. The survey results regarding parents’ own vaccination in adulthood revealed that 37.7% of parents of children with IBD and 29.4% of parents in the control group had never undergone preventive vaccination as adults. Among those respondents who had been vaccinated at least once in adulthood, 12.6% reported adverse events following immunization (AEFI): specifically, 11 (10.4%) participants in the IBD group and 17 (16%) in the control group. Reported adverse events included: redness at the injection site in 8 (7.5%) respondents with IBD vs. 3 (2.8%) in the control group (*p* = 0.11); pruritus (itching) in 4 (3.8%) vs. 4 (3.7%), respectively (*p* = 0.98); and cough and/or difficulty breathing in 5 (4.7%) vs. 4 (3.7%), respectively (*p* = 0.701).

### 3.4. Vaccination of Children with IBD and Healthy Children

In addition to questions regarding attitudes toward vaccination and their own vaccination history, respondents were asked whether they had vaccinated their children under the age of 6 (the age by which the primary vaccination schedule is typically completed according to the National Immunization Schedule). The survey showed that 102 (93.6%) respondents in the IBD group and 100 (93.4%) in the control group (*p* = 0.96) had vaccinated their children in the preschool years, either in accordance with the standard immunization schedule or following an individual schedule. Participants who refused vaccination were asked to specify the reasons (selecting one or more) for not vaccinating their children in the preschool years. The main reasons for refusal are presented in Table 2. Among the surveyed parents, the most common reason for refusal (in both the IBD children and control groups) was medical exemptions (medical contraindications)—10% in the IBD group and 7.5% in the control group. Less frequently, parents cited distrust in the quality and composition of vaccines (3.7% in the IBD group and 3.8% in the control group). Fear of developing diseases allegedly caused by vaccination was reported by 1.8% of respondents in the IBD group and 2.8% in the control group. Allergic reactions to vaccines were noted by 0.94% of participants. It was clarified that by “allergic reactions,” parents referred to occurrences such as urticaria or choking attacks that had ever happened to their children in response to vaccine administration.

We also asked parents what adverse events they believed had occurred in their children following vaccination. Based on the respondents’ opinions, a spectrum of the main adverse events arising in response to vaccination was compiled (Table 3). As shown in Table 3, parents primarily identified skin rash (8.3% in the IBD group vs. 12.3% in the control group), itching at the injection site (3.7% vs. 7.5%, respectively), and fever (12.8% vs. 0.9%, respectively) as the main adverse events. Less frequently, respondents noted inflammation at the injection site (5.5% in the IBD group) and cough (3.7% in the control group). Other reported reasons included constipation, leg pain, lethargy, and general malaise, with an equal number of such responses in each group, respectively.

**Figure 2:**
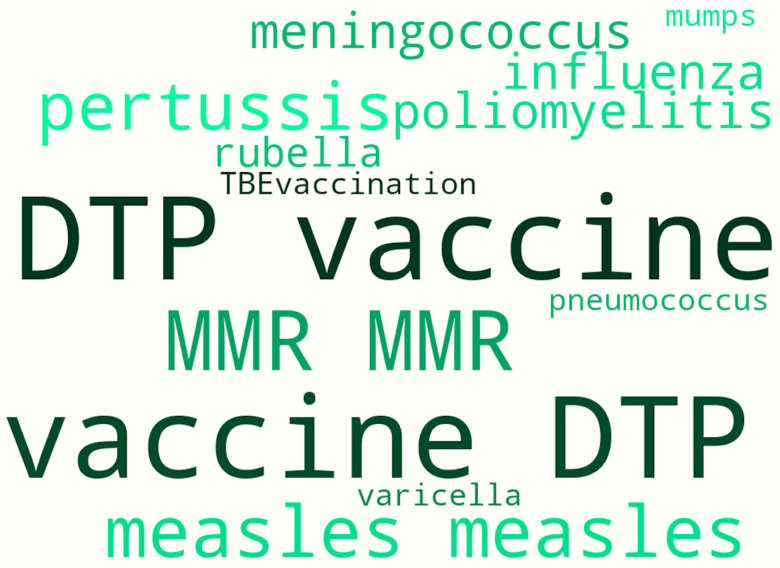
Vaccines most frequently associated with adverse events, according to respondents.

In addition to adverse events, we identified which vaccines, according to the surveyed parents, were most frequently associated with negative reactions. This question was open-ended (no pre-set options were provided), and all data were based solely on the respondents’ own knowledge and experience. Given the diversity of the responses, a “word cloud” map was generated to visualize all answers, weighted by the frequency of mentions from respondents in both groups. These data are presented in Figure 2. As shown in Figure 2, the DTP (diphtheria-tetanus-pertussis) vaccine holds the leading position in terms of mentions associated with adverse events (22 mentions; 10.2%). Additionally, “pertussis” was specifically mentioned 6 times (2.8%). Less frequently, parents mentioned “measles” and the combined MMR vaccine—11 (5.1%) and 8 (3.7%) instances, respectively. Meanwhile, “mumps” and “rubella” were cited quite rarely (1 (0.5%) and 2 (1%) cases, respectively). Among non-live/subunit vaccines, respondents pointed to meningococcal and pneumococcal vaccines in 3 (1.4%) and 1 (0.5%) case(s), respectively; influenza in 3 (1.4%) cases; and tick-borne encephalitis in 1 (0.5%) case. Chickenpox was mentioned in only 1 (0.5%) case. Adverse events related to the polio vaccine were reported in 3 (1.4%) cases.

### 3.5. Vaccination After the Onset of IBD

Additionally, among respondents in the IBD group, we conducted a survey regarding the vaccination of their children after the onset of the disease. Of all those surveyed, 24 (22%) parents reported that they continued vaccinating their children after the IBD diagnosis. Upon clarification, it was established that vaccination was administered against pneumococcal infection (a single dose among all respondents) and influenza (once a year for preventive purposes). The majority of respondents (85; 78%) categorically refused to continue further vaccination. However, more than half of the respondents (65; 59.6%) reported that they had explored the possibility of continuing vaccination. It is noteworthy that no parents with inflammatory bowel disease vaccinated their children following their diagnosis. Given the high rate of vaccination refusal after diagnosis, we analyzed potential factors influencing this decision (Table 4). We found no statistically significant differences in vaccination continuation rates based on the child’s sex (*p* = 0.6), disease phenotype (CD vs. UC, *p* = 0.4), primary activity (*p* = 0.53), or maternal education level (*p* = 0.35). However, parental attitude toward vaccination played a significant role (*p* = 0.012). A baseline positive attitude toward immunization was significantly associated with a higher likelihood of continuing vaccination after the onset of IBD. We also found a significant association between the parents’ own vaccination status and the decision to vaccinate their child (*p* = 0.02). Parents who underwent preventive vaccination in adulthood were significantly more likely to continue vaccinating their child after the IBD diagnosis (Table 4).

**Figure 3:**
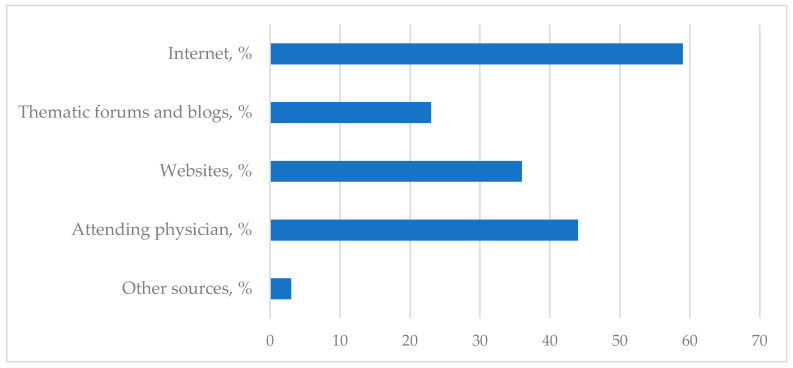
Main sources of information on the possibility of vaccination in children with IBD, according to respondents.

We clarified which information sources parents relied on most frequently when studying the feasibility of continuing vaccination (Figure 3). It was found that 43% of respondents had not sought information regarding further vaccination. Among those who did explore this issue, the internet was the primary source (59%), comprising websites (36%) and blogs/forums for parents of children with IBD (23%). For 44% of respondents, the attending physician was the main source of information. Only 3% of respondents reported independently searching for and reading professional literature and/or attending educational courses.

### 3.6. Factors Associated with Vaccination Continuation

To identify predictors of vaccination continuation after IBD onset, we performed a binary logistic regression analysis including maternal education and parental attitude as covariates. Maternal education level did not show a statistically significant association with the decision to continue vaccination (*p* > 0.05). However, baseline parental attitude was a significant predictor (*p* = 0.024). Parents who expressed skepticism were significantly less likely to continue vaccination compared to those with a positive attitude (OR 4.43; 95% CI 1.21–16.2).

## 4. Discussion

This study demonstrates the attitudes of parents of children with IBD toward vaccination. Currently, a rise in the incidence of vaccine-preventable diseases is being observed worldwide. For instance, between 2022 and 2023, 1274 cases of measles were registered in the USA [17]. In Japan, 2186 cases of rubella were confirmed among adolescents and adults in 2018 [18]. An increase in mumps incidence (up to 0.67 cases per 100,000 population in 2023) was noted among children aged one to four years in the USA [19]. Patients with IBD regularly receive immunosuppressive therapy, rendering them more susceptible to infectious diseases [3]. Patients on long-term high-level immunosuppression are at particular risk [20,21]. Kirchgesner et al. showed that among more than 190,000 patients with IBD, severe infections occurred in 8561 individuals, while opportunistic infections were recorded in 674 patients [5]. Moreover, patients receiving combination therapy consisting of anti-TNF agents and immunomodulators were at a higher risk of developing opportunistic infections compared to patients receiving anti-TNF monotherapy [5]. Furthermore, studies indicate that vaccination rates among patients with IBD often fall short of recommended targets. Authors from the ESPGHAN “Porto” group demonstrated that vaccination coverage at diagnosis was unsatisfactory for MMR (89.3%), *Haemophilus influenzae* type b (81.9%), meningococcal (23.5%), and pneumococcal (18.6%) infections, as well as varicella (5.9%) [22]. Full vaccination coverage was recorded in only 8.8% of the study participants [22]. Another study showed that vaccination compliance did not exceed 65% for any of the vaccines analyzed among 1722 patients with ulcerative colitis or Crohn’s disease [7]. Vaccination guidelines for patients with IBD have been established for a considerable time. According to the ECCO guidelines [23], routine vaccination with non-live vaccines is recommended for all patients, regardless of disease activity or current therapy. The authors place particular emphasis on additional vaccination of IBD patients against pneumococcal infection, influenza, human papillomavirus (HPV), and hepatitis B. Meanwhile, vaccination with live vaccines is recommended for patients on low-level immunosuppression or prior to the initiation of such therapy [23]. In the Canadian guidelines for immunization of patients with IBD, the authors emphasize the necessity of administering live vaccines to IBD patients, but only to those who are not receiving immunosuppressive therapy [24,25]. Nevertheless, despite the existence of numerous recommendations and evidence supporting the necessity and safety of vaccinating children with IBD, several barriers to implementing immunoprophylaxis in these patients persist.

### 4.1. Vaccination Barriers

One of the primary barriers to vaccinating patients with IBD, similar to all patients with systemic diseases, is the fear of disease exacerbation (flare-ups) and complications following immunization. Currently, there is no evidence linking vaccination to exacerbations of the underlying disease; vaccination with non-live vaccines is safe for patients receiving anti-TNF therapy [8,9]. A study by Longuet et al. showed that the main reasons for immunization refusal were fear of disease activation (3%) and fear of side effects (33%) [16]. In our study, 78% of surveyed parents refused further vaccination after the diagnosis of IBD was established. In a study by Huth et al., 19% of participants refused routine influenza vaccination due to fear of complications [26]. A survey of parents of IBD patients aged 10 to 25 years revealed that 40% of respondents were concerned about the potential worsening of their child’s health status as a result of vaccination [11]. Another barrier to immunization is patients’ lack of awareness regarding vaccination after the onset of IBD. A study by Ryu et al. showed that 87.1% of IBD patients were unaware of the possibility and necessity of continuing vaccination after diagnosis, and moreover, 79.4% had never received vaccination recommendations from their attending physicians [27]. The discrepancy between the high baseline positive attitude and the low actual vaccination rate indicates a phenomenon of “acquired hesitancy.” Parents do not oppose vaccination in principle but avoid it due to situational fears. This behavior is reinforced by the lack of proactive recommendations: without explicit permission and safety reassurance from their specialist, parents tend to perceive vaccination as an unnecessary risk. Among our patients, 43% of respondents had not sought information about the possibility of further vaccination. Furthermore, for our respondents, the internet was the primary source of information regarding vaccination feasibility. Over 59% of parents turned to websites (36%) as well as blogs and forums for parents of children with IBD (23%) for information. It is worth noting that the reliability of the information obtained by our respondents from the internet remains unknown. Similar findings are reported by Makarova et al., where a survey of parents of children with juvenile idiopathic arthritis (JIA) and IBD found that parents of children with IBD more frequently consulted internet forums for vaccination information [13]. Only 44% of parents in our study identified their attending gastroenterologist as a primary source of information. Here, we can identify another barrier to vaccination: the search for unverified and potentially unreliable information, which may shape an unfavorable perception of vaccination. For instance, a large-scale study conducted among the Russian population identified social networks and parenting forums as the main source of anti-vaccination sentiment [28]. Conversely, a survey of Korean IBD patients indicated that subscriptions to professional journals were the main source of information regarding IBD and vaccination options after disease onset [27].

### 4.2. Parental Attitudes and Misconceptions Regarding Vaccination

Despite the high rate of affirmative responses from respondents regarding early childhood vaccination (93.6% and 93.4% positive responses in the IBD and control groups, respectively), numerous prejudices surrounding immunization persist, shaping a negative perception alongside fears of complications and low awareness regarding vaccination feasibility post-IBD onset. We found that 30% of parents of children with IBD initially regarded vaccination with skepticism (distrust), and 7% held a negative attitude. In a recent survey of adult IBD patients, negative attitudes toward vaccination were expressed by 3.9% of respondents, while indifference was noted in 7.3% [29]. Despite the overwhelming majority of parents in our study being positively disposed toward vaccination, we established that approximately 30% of respondents in the IBD group and 37% in the control group had never undergone preventive vaccination themselves in adulthood. This represents a gap in their own immunization as well as potential awareness. According to official Russian statistics for 2024, the number of adults vaccinated against influenza reached 80%, which falls short of the target level of 85% [30]. In addition to gaps in their own immunization, we identified a lack of parental awareness regarding potential events following vaccination. Participants in both groups reported itching, pain, and swelling at the injection site, as well as fever in their children following vaccination. These events do not constitute complications or adverse events of vaccination but are normal physiological reactions to the administration of vaccine antigens [31]. However, insufficient parental knowledge about the possibility of such occurrences may contribute to their anxiety and predetermine a cautious or negative attitude toward future vaccinations. Notably, parents most frequently associated these symptoms with the DTP and MMR vaccines. Parents specifically lack clarity on the safety of non-live vaccines during immunosuppressive therapy and the distinction between expected immunogenic reactions (fever) and disease flares. We attribute this to the high antigenic load of the whole-cell DTP vaccine, which contains over 3000 antigens, thereby increasing its reactogenicity [32]; the MMR vaccine is a live vaccine and, like other live vaccines, elicits a more active immune response upon administration [33].

### 4.3. Strategies for Improvement

As previously mentioned, patients with IBD are at increased risk of infectious diseases and frequently have suboptimal vaccination rates. Our survey indicates that the primary necessity is to conduct educational work with parents of children with IBD. Parents often lack adequate knowledge regarding vaccination, a trend observed in both groups: parents of patients with IBD and parents of healthy children. We believe that attending gastroenterologists and pediatricians in specialized hospitals should organize educational schools for parents of children with IBD. This measure would enhance parental knowledge about vaccination. For instance, a study by Fleurier et al. demonstrated that educating patients with IBD resulted in increased vaccination rates across multiple indicators [14]. Similarly, research on educational schools for parents of patients with juvenile idiopathic arthritis (JIA), conducted by rheumatologists, showed a significant positive response from parents regarding improved awareness of vaccination opportunities [34]. Other studies have demonstrated the efficacy of inviting IBD patients for scheduled vaccination. According to Parker et al., distributing informational brochures to IBD patients about the benefits and safety of vaccination during visits to gastroenterologists and general practitioners increased influenza vaccination rates from 54% to 81%, and pneumococcal vaccination rates from 31% to 54% [15]. In addition to distributing informational leaflets and conducting popular science schools for parents, it is essential to hold educational seminars for physicians to improve their commitment to vaccination. A survey of gastroenterologists working in large hospitals and managing IBD patients revealed that only 30.9% of doctors requested vaccination records for IBD patients; 28% of respondents were convinced that the responsibility for immunizing IBD patients lies with primary care physicians [12]. Furthermore, 41% of doctors noted that poor coordination between gastroenterologists and primary care providers is a major predictor of incomplete vaccination in IBD patients [12]. Some studies have shown that physicians experience discomfort when making vaccination decisions for IBD patients unilaterally. The primary fear among doctors is related to concerns about potential IBD exacerbations [35]. It is particularly noteworthy that in Russia, there are no specifically developed regional or national methodological guidelines for vaccinating this patient group. In the absence of official guidelines, primary care physicians often face challenges in determining the appropriateness of continuing immunization for these patients. This underscores the critical need for the development and adoption of specific vaccination protocols for pediatric IBD. Referring children to a gastroenterologist prior to immunization could enhance parental confidence and facilitate individualized decision-making regarding vaccine eligibility. Furthermore, implementing targeted educational programs for parents would significantly improve their awareness and understanding of disease management, including the safety of preventive measures.

### 4.4. Limitations

Our study has several limitations. First, the work utilized a comparative design, which allows for the assessment of attitudes toward vaccination at a single point in time but precludes the possibility of tracking changes in parental opinions as the child grows or as the disease duration increases. Second, there are limitations related to the sample of respondents. The study was conducted at two specialized centers in a large metropolis, and all participants were urban residents. Furthermore, the overwhelming majority of parents (approximately 80%) had a higher education. This socio-demographic profile may not reflect the situation in rural areas or among populations with lower education and income levels, where barriers to vaccination may differ in nature. Third, the results are based on self-reported data. Information regarding vaccination history and, in particular, adverse events was obtained from parents’ accounts and was subject to their memory and subjective interpretation of symptoms. We did not use medical records to verify each case of described “reactions” or “complications” within the scope of this survey. Finally, although we compared demographic characteristics between groups, we did not perform a multivariate regression analysis to control for all potential confounding factors due to the limited sample size in certain subgroups, which may affect the generalizability of the findings.

## 5. Conclusions

Thus, a survey of a cohort of parents of children with IBD demonstrated that the low vaccination rate following diagnosis is not associated with inherent anti-vaccination sentiments among parents. The survey results indicate that prior to disease onset, vaccination coverage exceeded 93%, and the proportion of parents with negative attitudes did not exceed 7%, which is comparable to the responses of parents of healthy children. However, after the onset of IBD, only 22% of study participants continued vaccination. The key issue lies in the presence of unfounded fears regarding IBD exacerbation and vaccine side effects (particularly from live and whole-cell vaccines) in the absence of professional informational support. The identified knowledge deficit, concerning both child vaccination and parental revaccination, dictates the necessity of implementing new educational programs and strategies. Increasing parental awareness and the engagement of, primarily, primary care physicians as well as gastroenterologists in immunoprophylaxis issues remain the only effective tool for overcoming vaccination barriers.

## Figures and Tables

**Table 1:** Demographic and clinical characteristics of the study participants.

Characteristic	IBD Group	Control Group	*p*-Value
Number of parents interviewed, *n* (%)	109 (50.7)	106 (49.3)	n/a
Mothers			
Age, years, median (IQR)	35 (27–50)	30 (25–45)	0.763
Mother’s education, *n* (%)			
higher education (completed)	88 (81)	84 (79.2)	0.882
incomplete higher education	19 (17.4)	19 (17.9)	
incomplete secondary education	2 (1.9)	3 (2.9)	
Maternal attitude to vaccination, *n* (%)			
positive	68 (62)	77 (73)	0.27
skeptical	33 (30)	24 (23)	
negative	8 (7)	5 (5)	
Fathers			
Father’s education, *n* (%)			
higher education (completed)	73 (67)	79 (74.5)	0.002 *
incomplete higher education	35 (32)	18 (17)	
incomplete secondary education	1 (0.9)	9 (8.5)	
Children			
Age, years, median (IQR)	13 (10–16)	12 (9–15)	0.687
Sex, *n* (%)			
male	50 (45.9)	50 (47.2)	0.779
female	59 (54.1)	56 (52.8)	
Age at IBD onset, years, median (IQR)	11 (8–15)	n/a	n/a
Clinical phenotype, n (%)			
CD	55 (50.1)	n/a	n/a
UC	54 (49.9)	n/a	n/a
Primary Activity, *n* (%)			
School student (full-time)	84 (77)	81 (76)	0.91
School student (home-based)	5 (4.6)	n/a	n/a
College/University student	13 (11.9)	14 (13.2)	0.78
Preschooler (attends kindergarten)	2 (1.8)	10 (9.4)	0.02
Preschooler (does not attend)	5 (4.6)	1 (0.9)	0.11

Notes to Table 1. (*p* < 0.05 is considered statistically significant. Significant results are marked with an asterisk (*). n/a—no data available; IBD—Inflammatory bowel diseases; UC—ulcerative colitis; CD—Crohn’s disease; median (IQR)—median (interquartile range)).

**Table 2:** Major reasons for vaccination refusal among parents.

Reasons for Declining Vaccination	IBD Group	Control Group	OR; 95% CI	*p*-Value
Medical exemptions, *n* (%)	11 (10)	8 (7.5)	0.735; 0.283–1.91	0.53
Fear of developing diseases (neurological, autoimmune, oncological diseases), *n* (%)	2 (1.8)	3 (2.8)	1.46; 0.239–8.90	0.68
Distrust in vaccine quality and composition, *n* (%)	4 (3.7)	4 (3.8)	0.962; 0.234–3.95	0.98
Allergic reactions to vaccines, *n* (%)	1 (0.93)	1 (0.96)	0.963; 0.0594–15.6	0.98

Notes to Table 2. (*p* < 0.05 is considered statistically significant. IBD—Inflammatory bowel diseases; OR—odds ratio; 95% CI—95% Confidence Interval).

**Table 3:** Adverse reactions to vaccination, according to a survey of participants in the IBD and control groups.

Reasons for Vaccine Refusal	IBD Group	Control Group	OR; 95% CI	*p*-Value
Rash, *n* (%)	9 (8.3)	13 (12.3)	1.46; 0.596–3.58	0.41
Itching at the injection site, *n* (%)	4 (3.7)	8 (7.5)	2.02; 0.589–6.92	0.25
Fever, *n* (%)	14 (12.8)	1 (0.9)	0.06; 0.008–0.471	<0.001 *
Injection site inflammation, *n* (%)	6 (5.5)	0	n/a	0.029 *#
Cough, *n* (%)	0	4 (3.7)	n/a	0.057 #
Other reasons, *n* (%)	2 (1.8)	2 (1.9)	0.972; 0.134–7.03	0.98

Notes to Table 3. (*p* < 0.05 is considered statistically significant. Significant results are marked with an asterisk (*). The hash symbol (#) indicates the use of the exact Fisher criterion due to the zero values in the cells. OR—odds ratio; 95% CI—95% Confidence Interval; IBD—Inflammatory bowel disease. The category “Others” includes parental reports of leg pain, abdominal pain, and constipation).

**Table 4:** Factors associated with parental decision to continue or refuse vaccination after IBD onset.

Characteristic	Continued Vaccination	Refused Vaccination	*p*-Value
Number of patients with IBD *n* (%)	24 (22)	85 (78)	n/a
Clinical phenotype			
CD, *n* (%)	11 (45.8)	44 (51.8)	0.4
UC, *n (*%)	13 (54.2)	41 (48.2)	
Sex, *n* (%)			
male	12 (50)	45 (53)	0.6
female	12 (50)	40 (47)	
Primary Activity, *n* (%)			
school student (full-time)	19 (79.2)	65 (76.5)	0.009 *
school student (home-based)	2 (8.3)	3 (3.5)	<0.001 *
university student	2 (8.3)	5 (5.9)	<0.001 *
preschooler (attends kindergarten)	0	7 (8.2)	n/a
preschooler (does not attend)	1 (4.2)	5 (5.9)	<0.001 *
Maternal education, *n* (%)			
higher education	22 (91.7)	72 (84.7)	0.35
incomplete higher education	0	1 (1.2)	
incomplete secondary education	2 (8.3)	12 (14.1)	
Maternal attitude to vaccination, *n* (%)			
positive	22 (91.7)	50 (58.8)	0.012 *
skeptical	2 (8.3)	26 (30.6)	
negative	0	9 (10.6)	
Parental vaccination (adult), *n* (%)			
vaccinated	21 (87.5)	57 (67.1)	0.02 *
not vaccinated	3 (12.5)	28 (32.9)	

Notes to Table 4. (*p* < 0.05 is considered statistically significant. Significant results are marked with an asterisk (*). n/a—no data available; IBD—Inflammatory bowel diseases; UC—ulcerative colitis; CD—Crohn’s disease).

## Data Availability

The data presented in this study are available on request from the corresponding author. The data are not publicly available due to patient privacy concerns and Institutional data protection policies.

## Supplementary Materials

The following supporting information can be downloaded at: https://www.mdpi.com/article/10.3390/children13020238/s1. Section S1: General Data; Section S2: Vaccination and parental attitudes; Section S3: Child vaccination.

## References

[1] Dhaliwal J., Walters T.D., Mack D.R., Huynh H.Q., Jacobson K., Otley A.R., Debruyn J., El-Matary W., Deslandres C., Sherlock M.E. (2020). Phenotypic Variation in Paediatric Inflammatory Bowel Disease by Age: A Multicentre Prospective Inception Cohort Study of the Canadian Children IBD Network. J. Crohn’s Colitis.

[2] Levine A., Koletzko S., Turner D., Escher J.C., Cucchiara S., de Ridder L., Kolho K.L., Veres G., Russell R.K., Paerregaard A. (2014). ESPGHAN revised porto criteria for the diagnosis of inflammatory bowel disease in children and adolescents. J. Pediatr. Gastroenterol. Nutr..

[3] Shah B.B., Goenka M.K. (2020). A comprehensive review of vaccination in patients with inflammatory bowel diseases: An Indian perspective. Indian J. Gastroenterol..

[4] Papp K.A., Haraoui B., Kumar D., Marshall J.K., Bissonnette R., Bitton A., Bressler B., Gooderham M., Ho V., Jamal S. (2019). Vaccination Guidelines for Patients with Immune-mediated Disorders Taking Immunosuppressive Therapies: Executive Summary. J. Rheumatol..

[5] Kirchgesner J., Lemaitre M., Carrat F., Zureik M., Carbonnel F., Dray-Spira R. (2018). Risk of Serious and Opportunistic Infections Associated with Treatment of Inflammatory Bowel Diseases. Gastroenterology.

[6] Makarova E.Y., Alexeyeva E.I., Lomakina O.L., Gabrusskaya T.V., Ulanova N.B., Volkova N.L., Artemyeva A.A., Revnova M.O., Kostik M.M. (2024). Attitude towards vaccination among pediatric gastroenterologists and rheumatologists of several Russia regions after the anonymous online survey. Pediatr. G.N. Speransky.

[7] García-Serrano C., Mirada G., Marsal J.R., Ortega M., Sol J., Solano R., Artigues E.M., Estany P. (2020). Compliance with the guidelines on recommended immunization schedule in patients with inflammatory bowel disease: Implications on public health policies. BMC Public Health.

[8] Caldera F., Hillman L., Saha S., Wald A., Grimes I., Zhang Y., Sharpe A.R., Reichelderfer M., Hayney M.S. (2020). Immunogenicity of High Dose Influenza Vaccine for Patients with Inflammatory Bowel Disease on Anti-TNF Monotherapy: A Randomized Clinical Trial. Inflamm. Bowel Dis..

[9] Nguyen D.L., Nguyen E.T., Bechtold M.L. (2015). Effect of Immunosuppressive Therapies for the Treatment of Inflammatory Bowel Disease on Response to Routine Vaccinations: A Meta-Analysis. Dig. Dis. Sci..

[10] Ministry of Health of the Russian Federation Order No. 1122n of December 6, 2021 “On Approval of the National Calendar of Preventive Vaccinations and the Calendar of Preventive Vaccinations for Epidemic Indications”. http://publication.pravo.gov.ru/Document/View/0001202112200070.

[11] Holland K.J., Wilkinson T.A., Phipps E., Slaven J.E., Bennett W.E. (2020). Vaccination Rates and Family Barriers Among Children with Inflammatory Bowel Disease. Crohn’s Colitis 360.

[12] Lester R., Lu Y., Tung J. (2015). Survey of Immunization Practices in Patients with Inflammatory Bowel Disease Among Pediatric Gastroenterologists. J. Pediatr. Gastroenterol. Nutr..

[13] Makarova E., Khabirova A., Volkova N., Gabrusskaya T., Ulanova N., Sakhno L., Revnova M., Kostik M. (2023). Vaccination coverage in children with juvenile idiopathic arthritis, inflammatory bowel diseases, and healthy peers: Cross-sectional electronic survey data. World J. Clin. Pediatr..

[14] Fleurier A., Pelatan C., Willot S., Ginies J.L., Breton E., Bridoux L., Segura J.F., Chaillou E., Jobert A., Darviot E. (2015). Vaccination coverage of children with inflammatory bowel disease after an awareness campaign on the risk of infection. Dig. Liver Dis..

[15] Parker S., Chambers White L., Spangler C., Rosenblum J., Sweeney S., Homan E., Bensen S.P., Levy L.C., Dragnev M.C.C., Moskalenko-Locke K. (2013). A quality improvement project significantly increased the vaccination rate for immunosuppressed patients with IBD. Inflamm. Bowel Dis..

[16] Longuet R., Willot S., Giniès J.L., Pélatan C., Breton E., Segura J.F., Bridoux L., Le Henaff G., Cagnard B., Jobert A. (2014). Immunization status in children with inflammatory bowel disease. Eur. J. Pediatr..

[17] Moss W.J., Griffin D.E. (2024). What’s going on with measles?. J. Virol..

[18] Leung A.K.C., Hon K.L., Leong K.F. (2019). Rubella (German measles) revisited. Hong Kong Med. J..

[19] Tappe J., Leung J., Mathis A.D., Oliver S.E., Masters N.B. (2024). Characteristics of reported mumps cases in the United States: 2018–2023. Vaccine.

[20] Nicol S., Lawrence S., Jacobson K. (2017). Inadequate vaccination uptake in children receiving anti-TNF therapy for inflammatory bowel disease. Gastroenterology.

[21] deBruyn J.C.C., Soon I.S., Fonseca K., Feng S., Purtzki M., Goedhart C., Kuhn S., Vanderkooi O.G., Wrobel I. (2019). Serologic Status of Routine Childhood Vaccines, Cytomegalovirus, and Epstein-Barr Virus in Children with Inflammatory Bowel Disease. Inflamm. Bowel Dis..

[22] Martinelli M., Giugliano F.P., Strisciuglio C., Urbonas V., Serban D.E., Banaszkiewicz A., Assa A., Hojsak I., Lerchova T., Navas-López V.M. (2020). Vaccinations and Immunization Status in Pediatric Inflammatory Bowel Disease: A Multicenter Study from the Pediatric IBD Porto Group of the ESPGHAN. Inflamm. Bowel Dis..

[23] Kucharzik T., Ellul P., Greuter T., Rahier J.F., Verstockt B., Abreu C., Albuquerque A., Allocca M., Esteve M., Farraye F.A. (2021). ECCO Guidelines on the Prevention, Diagnosis, and Management of Infections in Inflammatory Bowel Disease. J. Crohn’s Colitis.

[24] Benchimol E.I., Tse F., Carroll M.W., deBruyn J.C., McNeil S.A., Pham-Huy A., Seow C.H., Barrett L.L., Bessissow T., Carman N. (2021). Canadian Association of Gastroenterology Clinical Practice Guideline for Immunizations in Patients with Inflammatory Bowel Disease (IBD)-Part 1: Live Vaccines. Gastroenterology.

[25] Jones J.L., Tse F., Carroll M.W., deBruyn J.C., McNeil S.A., Pham-Huy A., Seow C.H., Barrett L.L., Bessissow T., Carman N. (2021). Canadian Association of Gastroenterology Clinical Practice Guideline for Immunizations in Patients with Inflammatory Bowel Disease (IBD)-Part 2: Inactivated Vaccines. Gastroenterology.

[26] Huth K., Benchimol E.I., Aglipay M., Mack D.R. (2015). Strategies to Improve Influenza Vaccination in Pediatric Inflammatory Bowel Disease Through Education and Access. Inflamm. Bowel Dis..

[27] Ryu H.H., Chang K., Kim N., Lee H.S., Hwang S.W., Park S.H., Yang D.H., Byeon J.S., Myung S.J., Yang S.K. (2021). Insufficient vaccination and inadequate immunization rates among Korean patients with inflammatory bowel diseases. Medicine.

[28] Plakida A.V., Briko N.I., Namazova-Baranova L.S., Feldblyum I.V., Los’ N.A., Ivanova E.S. (2022). Increasing population adherence to vaccination: Evaluation and a systematic approach to implementation. Epidemiol. Vaccinal Prev..

[29] Costantino A., Michelon M., Noviello D., Macaluso F.S., Leone S., Bonaccorso N., Costantino C., Vecchi M., Caprioli F., AMICI Scientific Board (2023). Attitudes towards Vaccinations in a National Italian Cohort of Patients with Inflammatory Bowel Disease. Vaccines.

[30] Federal Service for Surveillance on Consumer Rights Protection and Human Wellbeing (Rospotrebnadzor) (2025). On the State of Sanitary and Epidemiological Wellbeing of the Population in the Russian Federation in 2024: State Report.

[31] MacDonald N.E., SAGE Working Group on Vaccine Hesitancy (2015). Vaccine hesitancy: Definition, scope and determinants. Vaccine.

[32] Kostinov M.P., Prutskova E.V., Cherdantsev A.P., Feiskhanova G.R., Kostinov A.M., Vlasenko A.E., Polishchuk V.B. (2020). Safety of pertussis vaccines for adolescents. J. Infectology.

[33] Kostinov M.P. (2020). The Combination Vaccine against Measles. Epidemiol. Vaccinal Prev..

[34] Lototskaya P.S., Sevostyanov V.K., Valieva S.I., Glazyrina A.A., Kurbanova S.K., Rassokha D.D., Balashov S.L., Erdes S.I., Zholobova E.S. (2024). Effectiveness of educational programs for parents of children with juvenile idiopathic arthritis: Initial results. Vopr. Prakt. Pediatr. Clin. Pract. Pediatr..

[35] Selby L., Hoellein A., Wilson J.F. (2011). Are primary care providers uncomfortable providing routine preventive care for inflammatory bowel disease patients?. Dig. Dis. Sci..

