# Supplementary material for: Parental Attitudes Towards Vaccination in Children with Inflammatory Bowel Disease: A Comparative Study
Source: Children (Basel). 2026 Feb 7;13(2):238. doi: 10.3390/children13020238 (PMC12939428; doi:10.3390/children13020238)
Supplement: Supplementary file 1 [file children-13-00238-s001.zip › children-4094280-supplementary.pdf]

**Section S1: General Data**	
Child's sex: (select one)	Male / female
Child's date of birth:	
What is your relationship to the child?	Mother<br>Father<br>Other (please specify)
Age of parent (legal guardian):	
What is your child's current primary activity?	Attends a preschool institution (kindergarten)<br>Does not attend a preschool institution (stays at home)<br>Attends school<br>Does not attend school (home-based education)
Parent's education level (mother)	Secondary education<br>Higher education<br>Incomplete higher education<br>Other
Parent's education level (father)	Secondary education<br>Higher education<br>Incomplete higher education<br>Other
**Section S2: Vaccination and parental attitudes**	
What is your attitude toward vaccination? (select one)	Positive (Vaccination is necessary to build immunity against infections)<br>Skeptical / with distrust (I think vaccination is needed, but I worry about the consequences)<br>Negative (I believe it can be dangerous to health)
Did you undergo vaccination in adulthood? (select one)	yes / no
Did you experience any negative (adverse) reactions to vaccine administration? (select one)	yes / no
If there were negative reactions, how did they manifest? (Select one or more; answer only if "Yes" to the previous question)	Skin rash<br>Lacrimation (watery eyes)<br>Itching (pruritus)<br>Cough<br>Difficulty breathing (dyspnea)<br>Anaphylaxis
**Section S3: Child vaccination**	
Did you vaccinate your child under the age of 6? (select one)	yes / no
If the child was not vaccinated from an early age, what were the reasons? (select one or more)	Allergic reactions to vaccines<br>Personal distrust of vaccines and their quality

	Medical exemptions (medical contraindications)<br>Fear of post-vaccination complications (development of infectious, neuropsychiatric, or autoimmune diseases)
Did your child ever experience negative reactions after vaccination? (select one)	yes / no
If there were negative reactions, how did they manifest? (Select one or more; answer only if "Yes" to the previous question)	Skin rash<br>Lacrimation (watery eyes)<br>Itching (pruritus)<br>Cough<br>Difficulty breathing (dyspnea)<br>Anaphylaxis
If there were negative reactions, to which vaccines (against which infections) did they occur?	
Was your child vaccinated after the primary diagnosis was established? (select one)	yes / no
If vaccination was continued, which vaccines were administered? (please specify)	
Did you discuss the possibility of vaccinating your child after the primary diagnosis was established with your attending physician? (select one)	yes / no
Did you look for information regarding vaccination after the onset of the disease?	yes / no
If yes, what were the sources? (select one or more; answer only if "Yes" to the previous question)	Internet websites<br>Parent forums<br>Internet bloggers<br>Consultation with the attending physician
